# Effect of purple corn extract on performance, antioxidant activity, egg quality, egg amino acid, and fatty acid profiles of laying hen

**DOI:** 10.3389/fvets.2022.1083842

**Published:** 2023-01-06

**Authors:** Jiaxuan Li, Di Zhou, Hui Li, Qingyuan Luo, Xu Wang, Jixiao Qin, Yiqing Xu, Qi Lu, Xingzhou Tian

**Affiliations:** ^1^Key Laboratory of Animal Genetics, Breeding and Reproduction in the Plateau Mountainous Region, Ministry of Education, College of Animal Science, Guizhou University, Guiyang, China; ^2^Testing Center for Livestock and Poultry Germplasm, Guizhou Agricultural and Rural Affairs Office, Guiyang, China; ^3^Institute of Animal Nutrition and Feed Science, Guizhou University, Guiyang, China

**Keywords:** anthocyanin, antioxidant activity, egg quality, amino acid, fatty acid, laying hen

## Abstract

The objective of this study was to investigate the effects of anthocyanin-rich purple corn extract (PCE) on performance, antioxidant potential, egg quality, egg amino acid and fatty acid profiles of laying hens during the late laying period. A total of 360 88-wk-old laying hens were randomly divided into 4 groups, and fed a basal diet (CON) or a basal diet supplemented with 120 (LP), 240 (MP), and 360 mg/kg (HP) PCE, respectively. No significant difference (*P* > 0.05) was observed in the ADFI or average egg weight among the groups. However, the mean feed to egg ratio was quadratically decreased (*P* < 0.05) in the LP and HP treatments. The mean TAC was linearly and quadratically increased (*P* < 0.05) in all PCE supplemented treatments. The mean SOD was linearly and quadratically increased (*P* < 0.05) in the HP treatment compared with CON and MP groups. The GPX was linearly and quadratically lower in the HP treatment compared to the CON and LP groups. Differently, the MDA was linearly and quadratically lower (*P* < 0.05) in the PCE treatments compared with the CON. The eggshell thickness value in MP and HP treatments were linearly and quadratically higher (*P* < 0.05) than that of the CON and LP groups. Hens fed PCE was linearly and quadratically increased (*P* < 0.05) most individual amino acids, essential amino acid and umami amino acid profiles in egg. The PCE treatments showed linearly and quadratic (*P* < 0.05) effect on the myristoleate, heptadecenoic acid, elaidic acid, eicosenoic acid, heneicosanoic acid, and eicosatrienoic acid concentrations. Moreover, dietary supplementation of PCE was quadratically increased egg stearic acid, oleic acid, arachidic acid, linolenic acid methyl ester, arachidonic acid, diphenylamine, docosahexaenoic acid, monounsaturated fatty acid, and polyunsaturated fatty acid compared to the CON. Therefore, dietary anthocyanin-rich PCE can enhance plasma antioxidant potential, is beneficial to egg production, and improves amino acids and fatty acids in hen eggs during the late laying period.

## 1. Introduction

Currently, the residues of antibiotics and drug resistance are becoming increasingly concerning, and green and safe poultry products have been increasingly welcomed by consumers (1). In recent years, many countries including China prohibited the use of antibiotics as feed additives. Thus, finding a safe substitute has been imperative. The plant extract is an excellent natural substitution for antibiotics because its addition to the diet could improve the growth, gut health, and improve performance and egg quality of chickens (2, 3). These reports have indicated that the secondary metabolites of plants can promote the growth of animals, enhance immunity, resist bacteria and disease, and improve the quality of animal products (4). Therefore, plant extracts are promising green feed additives that can replace antibiotics.

Anthocyanins are plant secondary metabolites that have excellent antioxidative capacity and antibacterial properties, which as natural antioxidants, have potential benefits for livestock and poultry production (5). Anthocyanin has strong antioxidant, immune regulation and anti-inflammation effects, which may improve the immune function and antioxidant effects of the animal, alleviating the breeding process of a variety of stress responses (6). This is because anthocyanins can provide hydrogen atoms for phenols and trap free radicals, thereby stabilizing compounds through various resonant forms, and showing high antioxidant activity (7). Hence, anthocyanins have strong antioxidative and radical scavenging activities and are a source of an attractive natural antioxidant (8). Kaya et al. (9) found that the addition of anthocyanin-rich grape seed extract could improve egg haugh units of laying hens. Sabet et al. (10) found that anthocyanin-rich sour tea (*Hibiscus Sabdariffa*) plant extract did not affect functional or immune parameters but did improve the plasma antioxidant status in laying hens.

Currently, eggs are an important component of human food, and thus can be enriched with antioxidants through manipulation of poultry feed (11). Various studies have showed that dietary supplementation with anthocyanin plant extract can improve the quality of egg production in poultry (12, 13). However, in the late laying period, laying hens are prone to convert excess nutrients into fat, causing imbalance of lipid oxidation and reducing antioxidant function, and leading to the decrease of performance (14). Additionally, purple corn is rich in anthocyanin, which may be an important source of anthocyanin extraction and has wide development and application prospects (15). However, reports about the role and mechanism of anthocyanins in laying hens during the late laying period are relatively rare. We hypothesized that the feeding of purple corn extract (PCE) can increase antioxidant activities and improve egg production, amino acids and fatty acids in hen eggs. Accordingly, the current study observed the effects of anthocyanins from PCE on the performance, plasma antioxidant activity, egg production, amino acids and fatty acids in egg of Chishui black-bone laying hens during the late laying period.

## 2. Materials and methods

### 2.1. Animals, diets, and experimental design

Chishui black-bone hen is one of the famous local poultry breeds in the Guizhou Province of China (16). Moreover, the feeding trial was carried out at the Guizhou Zhuxiang Chicken Breeding Co. Ltd., Chishui, China (28.590337 N, 105.697472 E). Amer et al. (17) demonstrated that the inclusion of 200 and 400 mg/kg anthocyanin-rich roselle, *Hibiscus sabdariffa* L. extract could improve antioxidant activity in chicken. In this study, 360 healthy and similar body weight (BW 1,728 ± 147 g, mean±standard deviation). Chishui black bone hens from 88 weeks during the late laying period were used. All hens were randomly allocated into four groups consisting of a basal diet (CON) or the basal diet supplemented with 120 (LP), 240 (MP), and 360 mg/kg (HP) anthocyanin-rich PCE. Each group had six replicates, and each replicate had 15 laying hens. The hen housed was kept at a cage, and each cage (0.076 m3) was kept at 3 hens. All experimental hens were under the same conditions with 16 h/d light, 21°C temperature and 45~65% humidity.

The PCE (Nanjing Herd Source Biotechnology Co., Ltd., Nanjing, China), a commercial extract, had a total anthocyanin concentration of 2,619 μg/g according to our previous study (18). The PCE was first mixed with chopped concentrate, then mixed with roughage to prepare basal diet. There was 14 d a preparation period prior to a 60 d formal experimental period of the feeding trial. Feed and water were provided *ad libitum* during the whole period. The nutritional requirements of laying hens were determined according to the farm requirements and the Chinese Standard for the Feeding Standard of chickens (NY/T33-2004) (19). The chemical compositions for dry matter (930.15), crude protein (988.05), calcium (927.02), and phosphorus (964.06) of feed were analyzed as per the method of the AOAC (20). The chemical composition and nutrition composition of basal diet as shown in Table 1.

**Table 1:** The chemical composition and nutrient levels of basal diet.

**Ingredients**,	**Content**	**Nutrient levels**,	**Content**
**% of fed basis**		**% of DM**	
Corn	61.50	Dry matter, % of the as-fed diet	92.59
Soybean meal	26.19	Crude protein	16.26
Soybean oil	1.05	Metabolizable energy, MJ/kg	12.22
Limestone	7.86	Calcium	3.23
Fishmeal	0.10	Total phosphorus	0.45
NaCl	0.30	Available phosphorus	0.19
Premix	3.00	Lysine	0.87
Total	100	Methionine	0.42
		Methionin+cysteine	0.71

Provided per kilogram: vitamin A, 330000 IU; vitamin D_3_, 133500 IU; vitamin E, 850 IU; vitamin B_1_, 70 mg; vitamin B_2_, 200 mg; vitamin B_6_, 135 mg; vitamin B_12_, 0.8 mg; vitamin K_3_, 85 mg; nicotinamide, 1,200 mg; pantothenic acid, 350 mg; biotin, 9 mg; choline chloride, 12,000 mg; Cu, 340 mg; Fe, 2,000 mg; Mn, 700 mg; Zn, 2,700 mg; Se, 12 mg. Metabolizable energy was calculated value according to the feed database in China (2018).

### 2.2. Performance

During the entire experimental period, the daily feed amount and surplus were collected to calculate average daily feed intake (ADFI). The daily egg yield and egg weight were recorded to calculate average egg weight (g) = total daily weight/total egg weight; moreover, laying rate per hen-day (HD, %) = (laying number/layer number) × 100, and feed to egg ratio = total feed intake (g)/total egg weight (g).

### 2.3. Plasma antioxidant activity parameters

Two hens from each replicate for a total of twelve hens per group at 20, 40, and 60 d before feeding were randomly selected, blood (about 5 mL) was obtained from the inferior wing vein by a negative pressure vacuum tube with 12.5 IU/mL heparin sodium (Nanchang Ganda Medical Instrument Co., Ltd., Nanchang, China). Blood samples were centrifuged (KJH80-2, Jiangsu KangJianhua Medical Supplies Co., Ltd., Jiangsu, China) at 4,000 × *g* for 15 min, and the plasma was collected and kept at a −80°C fridge for further analysis. Total antioxidant capacity (TAC, A015-1), superoxide dismutase (SOD, A001-3), glutathione peroxidase (GPX, A005), catalase (CAT, A007-1), and malondialdehyde (MDA, A003-1) were determined, and all kids purchased from Nanjing Jiangcheng Bioengineering Institute (Nanjing, China).

### 2.4. Egg quality

Two hens from each replicate for a total of twelve hens per group at 20 d, 40 d and 60 d were randomly selected, and egg weight, albumen height, yolk weight, yolk color, and haugh unit were analyzed by an egg quality meter (EA-01, ORKA, Israel). Moreover, eggshell strength was analyzed by an eggshell strength tester (KQ-1A, Nannong Animal Husbandry Technology Co., Ltd., Beijing, China). Moreover, the egg shape index and the thickness of the eggshell were determined using cursor calipers (3 V, MASTERPROOF professional TOOL, Germany).

### 2.5. Egg amino acids and fatty acids

Two eggs from each replicate and a total of twelve eggs per group at the end of experimental period were randomly selected, and transferred into a vacuum freeze dryer (LYOQUEST-85 Plus, Bole Technology, Spain). The condenser was set at −80°C, and the vacuum pump was set to 0.000 m Bar. Next, the egg samples were ground by a high-speed grinder after 72 freeze-drying cycles. Amino acids and fatty acids were detected according to our previous study (16). Briefly, amino acid was analyzed using an automatic amino acid analyser (Biochrom 30, Biochrom Ltd., Cambridge, the United Kingdom). The amino acid analyser conditions were as follows: the chromatographic column was a sulfonic acid cation resin; detection wavelengths were 570 and 440 nm, respectively; individual amino acid was determined using peak area according to external standard method. For fatty acid, approximately 1 g of sample was weighed for hydrolysis; then, the fat extract was obtained, fat saponification and fatty acid esterification were processed, and the upper solution was collected and kept in a sample bottle. The individual fatty acids were detected by an Agilent 6,890 gas chromatograph (Agilent Technologies Co. Ltd., Palo Alto, USA). The gas chromatograph conditions were as follows: the injection volume was 1.0 μL; capillary column was a polar stationary phase of 100 m × 0.25 mm × 0.2 μm polydicyanide siloxane; 270°C of injector temperature; 280°C of detector temperature. The individual fatty acids were calculated using the peak area normalization method.

### 2.6. Statistical analysis

All raw data were recorded with Microsoft Excel 2010. All observations were analyzed by the general linear models procedure using in Statistical Product and Service Solutions 20.0 software (SPSS Inc., Chicago, Illinois, USA) with the least square mean noted by Tukey's test. All variables were tested for linear and quadratic effects, and the level of significance was assessed at *P* < 0.05.

## 3. Results

### 3.1. Performance

The mortality of hens did not differ (*P* > 0.05) throughout the feeding experimental period (data not shown; Table 2). No significant difference (*P* > 0.05) was observed in the ADFI or average egg weight among the groups during the entire experimental period. Compared to the CON, dietary supplementation of PCE exhibited linear and quadratic effect *(P* < 0.05) on the HD, with the highest values noted at HP treatment. Moreover, the feed to egg ratio was not significantly (*P* > 0.05) different in all groups from d 21 to 40 and d 21 to 40 except for the d 41 to 60, which was quadratically decreased relative to the CON group. In addition, the mean feed to egg ratio was quadratically decreased in the LP and HP treatments.

**Table 2:** Effect of purple corn extract on performance in laying hen.

**Items**	**PCE supplemental levels (mg/kg)**				**SEM**	***P-*** **Value**	
	**0**	**120**	**240**	**360**		**Linear**	**Quadratic**
**90–93 weeks**							
ADFI, g	95.22	96.14	93.67	95.96	1.99	0.976	0.942
Average egg weight, g	52.75	53.26	53.42	53.26	0.35	0.280	0.350
Laying rate per hen-day, %	31.32	38.23	33.95	41.65	0.03	0.001	0.005
Feed to egg ratio	6.23	4.95	5.68	4.55	0.30	0.651	0.694
**93–96 weeks**							
ADFI, g	101.12	100.71	97.72	99.13	2.01	0.018	0.035
Average egg weight, g	55.39	55.13	55.32	54.61	0.41	0.039	0.074
Laying rate per hen-day, %	41.02	44.19	42.83	51.79	0.02	0.000	0.000
Feed to egg ratio	4.53	4.26	4.30	3.67	0.12	0.229	0.177
**96–99 weeks**							
ADFI, g	97.73	97.82	93.67	97.76	2.02	0.336	0.061
Average egg weight, g	56.61	57.4	57.63	55.37	0.47	0.421	0.209
Laying rate per hen-day, %	43.25	42.59	41.61	48.89	0.02	0.004	0.000
Feed to egg ratio	4.20	4.21	4.09	3.71	0.15	0.795	0.040
**Mean**							
ADFI, g	98.02	98.22	96.56	97.62	0.75	0.224	0.161
Average egg weight, g	54.76	54.62	54.42	53.68	0.49	0.458	0.164
Laying rate per hen-day, %	38.53	41.67	39.46	47.45	0.08	0.000	0.000
Feed to egg ratio	4.99	4.47	4.69	3.98	0.14	0.574	0.040

ADFI, average daily feed intake; SEM, standard error of mean.

### 3.2. Antioxidant activity parameters

For day 20, there was no differences (*P* > 0.05) among groups in terms of TAC, SOD, and CAT concentrations (Table 3). The GPX was linearly and quadratically decreased (*P* < 0.05) in the HP treatment. For day 40, the TAC was linearly and quadratically increased (*P* < 0.05) in MP and HP treatments. The SOD was not significantly (*P* > 0.05) different between all groups. The GPX was linearly and quadratically decreased (*P* < 0.05) in the HP treatment. For day 60, the TAC and SOD were linearly and quadratically increased (*P* < 0.05) in the HP treatment. The mean TAC was linearly and quadratically increased (*P* < 0.05) in all PCE supplemented treatments. The mean SOD was linearly and quadratically increased (*P* < 0.05) in the HP treatment compared with CON and MP groups. The mean GPX was linearly and quadratically lower in the HP compared to the CON and LP groups. During the whole experimental period, no significant (*P* > 0.05) differences were found in the plasma CAT among the four groups; whereas the MDA was linearly and quadratically lower (*P* < 0.05) in the PCE treatments compared with the CON group.

**Table 3:** Effect of purple corn extract on plasma antioxidant activity in laying hen.

**Items**	**PCE supplemental levels (mg/kg)**				**SEM**	***P-*** **value**	
	**0**	**120**	**240**	**360**		**Linear**	**Quadratic**
**93-week**							
TAC, U/mL	5.49	6.43	6.08	6.24	0.57	0.409	0.538
SOD, U/mL	18.15	19.40	18.34	18.82	0.51	0.711	0.753
GPX, U/mL	224.03	237.50	209.30	197.58	5.95	0.000	0.000
CAT, U/mL	4.14	4.48	4.35	4.73	0.23	0.170	0.389
MDA, nmol/mL	11.21	7.64	6.99	7.02	0.54	0.000	0.000
**96-week**							
TAC, U/mL	5.54	5.75	8.77	8.31	0.56	0.000	0.000
SOD, U/mL	19.53	19.49	20.82	20.35	0.39	0.062	0.064
GPX, U/mL	162.90	165.39	157.75	144.57	1.74	0.000	0.000
CAT, U/mL	6.22	6.23	5.81	5.65	0.25	0.060	0.114
MDA, nmol/mL	9.00	7.32	7.09	7.34	0.48	0.009	0.003
**99-week**							
TAC, U/mL	3.38	7.47	5.19	8.11	0.74	0.034	0.100
SOD, U/mL	19.04	18.92	18.58	20.90	0.49	0.022	0.002
GPX, U/mL	187.21	200.78	183.04	188.76	4.07	0.482	0.500
CAT, U/mL	4.26	4.26	4.08	4.64	0.22	0.364	0.315
MDA, nmol/mL	10.66	7.38	7.92	7.72	0.60	0.013	0.005
**Mean**							
TAC, U/mL	4.88	6.41	6.60	7.46	0.39	0.000	0.000
SOD, U/mL	19.01	19.27	19.00	20.04	0.29	0.007	0.019
GPX, U/mL	192.19	202.51	185.69	176.97	3.45	0.000	0.000
CAT, U/mL	4.86	4.98	4.90	5.02	0.16	0.956	0.902
MDA, nmol/mL	10.11	7.40	7.26	7.28	0.31	0.000	0.000

TAC, total antioxidant capacity; SOD, superoxide dismutase; MDA, malondialdehyde; GPX, glutathione peroxidase; CAT, catalase; SEM, standard error of mean.

### 3.3. Egg quality

No significant difference (*P* > 0.05) was detected in egg shape index, eggshell strength, yolk weight, albumen height, yolk color, and haugh unit among the four groups during the entire experimental period (Table 4). The eggshell thickness was quadratically decreased (*P* < 0.05) in the LP and MP treatments at day 40. The eggshell thickness was linearly and quadratically increased (*P* < 0.05) in all PCE supplemented treatments at day 60. Moreover, the eggshell thickness value in MP and HP treatments were linearly and quadratically higher (*P* < 0.05) than that of the CON and LP groups.

**Table 4:** Effect of purple corn extract on egg quality in laying hen.

**Items**	**PCE supplemental levels (mg/kg)**				**SEM**	***P-*** **value**	
	**0**	**120**	**240**	**360**		**Linear**	**Quadratic**
**90–93 weeks**							
Egg-shaped index	1.31	1.30	1.31	1.32	0.02	0.454	0.688
Eggshell strength, kgf/cm^2^	42.83	41.14	38.49	38.21	2.63	0.116	0.281
Eggshell thickness, mm	0.27	0.25	0.29	0.27	0.01	0.197	0.378
Yolk weight, g	17.36	17.55	16.91	17.35	0.34	0.646	0.835
Albumen height, mm	4.03	4.16	4.11	4.41	0.21	0.243	0.471
Yolk color	6.92	7.92	7.82	7.33	0.40	0.525	0.144
Haugh unit	57.46	61.41	64.01	60.43	2.66	0.334	0.227
**93–96 weeks**							
Egg-shaped index	1.36	1.36	1.33	1.32	0.01	0.083	0.094
Eggshell strength, kgf/cm^2^	48.27	43.06	42.64	44.36	2.82	0.293	0.231
Eggshell thickness, mm	0.33	0.29	0.29	0.30	0.01	0.053	0.029
Yolk weight, g	17.64	18.72	18.38	17.59	0.39	0.770	0.135
Albumen height, mm	3.76	4.20	3.76	3.88	0.18	0.905	0.663
Yolk color	6.33	6.64	6.18	7.67	0.44	0.077	0.086
Haugh unit	58.72	62.61	57.78	59.00	1.52	0.534	0.535
**96–99 weeks**							
Egg-shaped index	1.33	1.31	1.30	1.31	0.01	0.192	0.183
Eggshell strength, kgf/cm^2^	45.71	42.73	48.31	42.61	1.90	0.643	0.674
Eggshell thickness, mm	0.18	0.25	0.28	0.29	0.01	0.000	0.000
Yolk weight, g	17.30	18.46	18.29	18.01	0.43	0.310	0.142
Albumen height, mm	4.06	4.92	3.93	4.44	0.26	0.901	0.812
Yolk color	6.00	5.75	6.42	6.17	0.48	0.583	0.583
Haugh unit	57.64	68.31	54.40	64.25	3.44	0.724	0.935
**Mean**							
Egg-shaped index	1.33	1.32	1.31	1.32	0.01	0.187	0.330
Eggshell strength, kgf/cm^2^	45.68	42.38	42.68	41.90	1.46	0.071	0.154
Eggshell thickness, mm	0.26	0.26	0.29	0.29	0.01	0.002	0.009
Yolk weight, g	17.43	18.22	17.82	17.64	0.23	0.794	0.072
Albumen height, mm	3.95	4.44	3.93	4.24	0.13	0.513	0.657
Yolk color	6.42	6.77	6.79	7.06	0.27	0.106	0.267
Haugh unit	57.89	64.25	58.65	61.29	1.59	0.527	0.417

SEM, standard error of mean.

### 3.4. Egg amino acids

The inclusion of PCE was linearly and quadratically increased (*P* < 0.05) most individual amino acid (including aspartate, threonine, serine, glutamate, glycine, alanine, valine, isoleucine, leucine, threonine, histidine, lysine (Lys), arginine, and proline) concentrations in eggs compared to the without PCE group (Table 5). Dietary PCE addition had no significant effect (*P* > 0.05) on the egg methionine content among four groups. The phenylalanine was quadratically increased (*P* < 0.05) in the PCE treatments relative to the CON group. In addition, hens fed PCE was linearly and quadratically increased in essential amino acid (EAA) and umami amino acid (UAA) profiles in egg.

**Table 5:** Effect of purple corn extract on egg amino acid profiles of 99-week-old laying hens.

**Items (%)**	**PCE supplemental levels (mg/kg)**				**SEM**	***P-*** **value**	
	**0**	**120**	**240**	**360**		**Linear**	**Quadratic**
Asparagine	4.62	4.85	4.93	4.88	0.05	0.011	0.002
Threonine	2.36	2.44	2.52	2.48	0.03	0.011	0.005
Serine	3.48	3.59	3.68	3.66	0.04	0.005	0.004
Glutamate	5.69	5.99	6.05	5.97	0.06	0.031	0.002
Glycine	1.54	1.61	1.65	1.62	0.02	0.017	0.002
Alanine	2.50	2.61	2.66	2.62	0.02	0.019	0.003
Valine	2.91	3.07	3.13	3.09	0.03	0.010	0.001
Methionine	1.40	1.47	0.91	1.39	0.02	0.341	0.197
Isoleucine	2.38	2.41	2.46	2.44	0.03	0.008	0.003
Leucine	3.87	4.05	4.15	4.12	0.04	0.004	0.001
Threonine	1.99	2.09	2.13	2.11	0.03	0.016	0.009
Phenylalanine	2.99	3.13	3.17	3.10	0.03	0.074	0.004
Histidine	1.01	1.07	1.09	1.07	0.01	0.018	0.003
Lysine	3.32	3.48	3.50	3.51	0.03	0.009	0.005
Argnine	2.97	3.12	3.18	3.16	0.03	0.005	0.001
Proline	1.67	1.76	1.83	1.80	0.02	0.004	0.001
EAA	18.77	19.66	20.02	19.81	0.19	0.027	0.040
UAA	19.33	20.30	20.58	20.31	0.20	0.022	0.003

EAA, essential amino acids; UAA, umami amino acids; SEM, standard error of mean. EAA, threonine+valine+methionine+isoleucine+leucine+phenylalanine+lysine; UAA, asparagine+glutamate+glycine+alanine+threonine+phenylalanine.

### 3.5. Egg fatty acids

There were no significant differences (*P* > 0.05) for lauric acid (C12:0), myristic acid (C14:0), myristoleate (C14:1), palmitate (C16:0), palmitoleate (C16:1), margaric acid (C17:0), linolelaidic acid (C18:2n6t), linoleic acid (C18:2n6c), methyl linolenate (C18:3n6), eicosadienoic acid (C20:2), behenic acid (C22:0), erucic acid (C22:1n9), and SFA in egg among all the groups (Table 6). The PCE treatments showed linearly and quadratic (*P* < 0.05) effect on the myristoleate (C15:0), heptadecenoic acid (C17:1), elaidic acid (C18:1n9t), eicosenoic acid (C20:1n9), heneicosanoic acid (C21:0), eicosatrienoic acid (C20:3n6) concentrations. Moreover, dietary supplementation of PCE was quadratically increased egg stearic acid (C18:0), oleic acid (18:1n9c), arachidic acid (C20:0), linolenic acid methyl ester (C18:3n3), arachidonic acid (C20:4n6), diphenylamine (DPA), docosahexaenoic acid (DHA), monounsaturated fatty acid, and polyunsaturated fatty acid (PUFA) compared to the CON group.

**Table 6:** Effect of purple corn extract on egg fatty acid profiles of 99-week-old laying hens.

**Items (%)**	**PCE supplemental levels (%)**				**SEM**	***P-*** **value**	
	**0**	**120**	**240**	**360**		**Linear**	**Quadratic**
Lauric acid, C12:0	0.007	0.009	0.009	0.011	0.00	0.240	0.517
Myristic acid, C14:0	0.445	0.495	0.439	0.439	0.00	0.256	0.097
Myristoleate, C14:1	0.118	0.148	0.109	0.117	0.00	0.301	0.274
Myristoleate, C15:0	0.039	0.043	0.043	0.043	0.00	0.009	0.000
Palmitate, C16:0	26.533	27.411	26.020	26.603	0.03	0.406	0.649
Palmitoleate, C16:1	3.911	4.492	3.679	3.928	0.01	0.369	0.462
Margaric acid, C17:0	0.125	0.119	0.136	0.131	0.00	0.060	0.188
Heptadecenoic acid, C17:1	0.100	0.099	0.103	0.142	0.00	0.001	0.000
Stearic acid, C18:0	8.055	7.693	7.871	8.128	0.02	0.417	0.000
Elaidic acid, C18:1n9t	0.039	0.045	0.099	0.145	0.02	0.002	0.008
Oleic acid, C18:1n9c	47.972	46.231	47.197	47.871	0.08	0.746	0.002
Linolelaidic acid, C18:2n6t	0.013	0.028	0.017	0.011	0.01	0.554	0.215
Linoleic acid, C18:2n6c	10.882	10.897	12.038	10.337	0.01	0.782	0.050
Arachidic acid, C20:0	0.037	0.025	0.027	0.029	0.00	0.087	0.000
Methyl linolenate, C18:3n6	0.052	0.098	0.073	0.061	0.01	0.977	0.226
Eicosenoic acid, C20:1n9	0.014	0.303	0.287	0.272	0.01	0.011	0.000
Linolenic acid methyl ester, C18:3n3	0.219	0.256	0.248	0.234	0.00	0.350	0.000
Heneicosanoic acid, C21:0	0.016	0.017	0.047	0.041	0.00	0.001	0.003
Eicosadienoic acid, C20:2	0.113	0.109	0.121	0.104	0.00	0.389	0.128
Behenic acid, C22:0	0.084	0.093	0.070	0.080	0.00	0.110	0.295
Erucic acid, C22:1n9	0.016	0.012	0.020	0.026	0.00	0.054	0.070
Eicosatrienoic acid, C20:3n6	0.113	0.116	0.112	0.108	0.00	0.009	0.000
Arachidonic acid, C20:4n6	0.886	1.008	1.007	0.925	0.00	0.438	0.000
Diphenylamine, DPA	0.041	0.046	0.045	0.039	0.00	0.515	0.000
Docosahexaenoic acid, DHA, C22:6n3	0.154	0.207	0.182	0.176	0.00	0.456	0.000
SFA	35.341	35.906	34.661	35.504	0.04	0.563	0.764
MUFA	52.171	51.330	51.495	52.501	0.07	0.406	0.000
PUFA	12.473	12.764	13.844	11.994	0.02	0.856	0.012

SFA, total saturated fatty acid profiles; MUFA, total monounsaturated fatty acid profiles; PUFA, total polyunsaturated fatty acid profiles; SEM, standard error of mean.

## 4. Discussion

The free radicals in poultry are usually in homeostasis and have important biological functions under normal circumstances (21). However, free radicals accumulated and resulted in animals entering the oxidative status, reducing the resistance and production performance of poultry (22). Plant flavonoids could improve the reproductive capacity of animals by releasing hormones, such as growth hormone, in livestock and poultry (23). Additionally, animals receiving anthocyanin extract can enhance digestive enzyme activity, thus improving feed digestibility and affecting nutrient absorption (17). Hence, we found that PCE could improve the HD values, which might be related to anthocyanin improve reproductive capacity and nutrient absorption. Consistent with our results, Tufarelli et al. (24) found that the feeding of anthocyanin-rich dried grape pomace in a laying hen diet did not affect the ADFI, whereas it could improve egg production. Duenas et al. (25) suggested that broiler chickens receiving anthocyanin-rich cranberry extract had an improved feed conversion ratio relative to the control group.

Radical reactions in the body must be in dynamic balance. Oxidative injury induced by excess free radicals is an etiology of several diseases. As a high-yielding animal species, laying hens have a more vigorous metabolism, producing higher levels of reactive oxygen free radicals. Generally, the activity of antioxidant enzymes decreases, and free radicals accumulate, and thus negatively affecting health and production of laying hens (26). Anthocyanin-rich plants can improve antioxidant potential by decreasing thio-barbituric acid-reactive substance values in breast meat in broilers (27). In addition, plant flavonoids can decrease the production of proliferation, T-cell activation, and proinflammatory cytokines, and thus regulating the immune system in animals (28). Saleh et al. (29) concluded that dietary supplementation with anthocyanin-rich sorghum can modulate the abundances of the mRNAs of genes related to antioxidative properties, such as GPX and SOD, which were increased in chickens. Moreover, anthocyanin plants can relieve the inflammatory reaction by the expression of genes related to interleukin-1β and interleukin-6 in chickens, thus enhancing antioxidants in broiler chickens (30). Hence, we found that the inclusion of PCE in hen diets can increase the concentration of TAC, SOD, and GPX, as well as decrease the content of MDA in blood. This might be because anthocyanins can provide excess phenolic hydrogen atoms to free radicals and neutralize free radicals in the animal body (31); and anthocyanins can participate in the metabolism of phospholipids, arachidonic acid and protein phosphorylation to protect lipids from oxidative damage (32). Consistent with our findings, Wang et al. (33) showed that the addition of 100 mg/kg anthocyanin-rich bilberry extract could increase SOD and GPX, but it could decrease MDA of yellow-feathered chickens. Additionally, Reis (34) demonstrated that laying hens receiving anthocyanin-rich grape pomace flour could increase TAC, SOD and GPX in the serum compared to the control.

Generally, with the increase in the daily age of laying layers, the eggshell quality of eggs produced decreases (35). Free radicals accumulate in the body due to their long oxidative metabolism, which may also damage the formation of egg protein and reduce the protein haugh unit of the laying hens at the later laying period (36). Moreover, the decline in eggshell quality were decreases of estrogen levels, calcium absorption and metabolic capacity in late egg laying (37). Notably, the inclusion of phenolic-rich plant extract may regulate the intestinal microflora profile in chickens, and improving health condition (38). Of interest, anthocyanins and their metabolites can reduce pathogenic bacteria and improve beneficial bacteria (*Lactobacillus* and E*nterococcus*), improving animal health and increasing production performance in poultry (39). Thus, we found that laying hens fed a diet containing anthocyanin-rich PCE showed higher value of eggshell thickness in eggs, perhaps due to anthocyanin can improve the intestinal environment and liver SOD concentration in laying hens (40, 41). Furthermore, Kara et al. (42) demonstrated that supplementing laying hen diets with 4% anthocyanin-rich grape pomace has the potential to increase egg weight, improving the egg quality of 80-week-old 96 Bovans laying hens. In short, anthocyanin-rich PCE improved the nutritional value of eggs, which has a higher market value and is more conducive to future application in production.

The amino acid content of eggs is balanced, which is easily absorbed and utilized by consumers, and it is the most ideal source of high-quality protein in natural food (43). Dietary supplementation with natural antioxidants in animal diets could improve UAA concentrations because it can increase antioxidant potential and regulate related umami gene expression in the body (44). In the current research, we found that the feeding of anthocyanins from PCE could improve UAA concentrations in eggs compared to the CON group. This possibly due to purple corn anthocyanins might take part in the UAA signaling pathway in laying hens, resulting in the higher EAA and UAA content in eggs in Chishui black bone hens. The exact reasons remain unclear, and further observations are needed. Our observations are in agreement with Omar et al. (45), who showed that phenolic-rich onion extract could improve the amino acid ileal digestibility of amino acids in broiler chicken. Mishra et al. (46), who found that anthocyanins could enhance higher levels of antioxidant activity, and improve the concentrations of Lys and tryptophan in eggs in white leghorn layer chickens.

The PUFAs are important to the human body, and the intake of a certain amount of unsaturated fatty acids has a good regulatory effect on blood fat, vision and immune function. However, PUFA molecules could transform into hydroperoxides, conjugated dienes, and peroxy radicals (47). Anthocyanins have antioxidant properties, which can prevent unsaturated fatty acids on the cell membrane from being oxidized. Crescenti et al. (48) showed that anthocyanin-rich grape seed can promote related fatty acid absorption and beta-oxidation genes overexpression, which can subsequently ameliorate obesity and metabolic disorders. Thus, MP and HP treatments increased UFA concentration in eggs, perhaps because anthocyanins not only exhibit a higher oxygen radical absorbance capacity; but also can provide hydrogen donors to the lipid free radicals, and thus resulting in the inhibition of lipid peroxidation (49–51). Consistent with our observations, Untea et al. (52) found that anthocyanin-rich bilberry leaves could improve egg UFA profiles (C18:2n6, C18:3n6, C20:3n6 and total n-6 fatty acids) in laying hens.

## 5. Conclusions

The results of the current research suggest that dietary supplementation with PCE can enhance plasma antioxidation ability, increase egg production, and improve egg amino acid and fatty acid profiles during the late laying period of laying hens.

## Data availability statement

The original contributions presented in the study are included in the article/supplementary material, further inquiries can be directed to the corresponding author.

## Ethics statement

The animal study was reviewed and all experimental animal care procedures were approved by the Rules of Animal Welfare and Experimental Animal Ethics of Guizhou University (EAE-GZU-2021-P017), Guizhou, China.

## Author contributions

JL conducted writing–original draft, data curation, methodology, and software. DZ and HL conducted resources, investigation, and supervision. QLuo, XW, JQ, and YX conducted resources and validation. QLu contributed by investigation. XT contributed by resources, writing–review and editing, software, and project administration. All authors contributed to the article and approved the submitted version.
